# Prevention of chronic disease using vitamins—a case study of the vitamin D and cardiovascular disease hypothesis using evidence from randomised controlled and prospective cohort studies

**DOI:** 10.1007/s00394-025-03706-w

**Published:** 2025-05-31

**Authors:** Mairead Kiely, Lorraine Brennan, Travis Dunlop, Gerard Tate, Jayne V. Woodside, Diego Moretti, on behalf of Working Group 1 of the Federation of European Nutrition Societies (FENS) Presidential Activity

**Affiliations:** 1 Cork Centre for Vitamin D and Nutrition Research, School of Food and Nutritional Sciences, University College Cork, Cork, T12 WE28 Ireland; 2 School of Agriculture and Food Science, UCD Institute of Food and Health, UCD Conway Institute, University College Dublin, Dublin, D04 V1 W8 Ireland; 3 School of Medicine, Dentistry and Biomedical Sciences, Queen’s University Belfast, Belfast, BT9 5DL UK; 4 Department of Health, Swiss Distance University of Applied Sciences (FFHS), University of Applied Sciences of South Switzerland (SUPSI), 8005 Zurich, Switzerland

**Keywords:** Nutrition science, Randomised controlled trials, Vitamins, Vitamin D, Cardiovascular disease, Bradford hill

## Abstract

**Purpose:**

The hypothesis that vitamin supplementation may prevent cardiovascular disease (CVD) has been supported by compelling mechanistic and observational data. However, most large-scale randomized controlled trials (RCT) of vitamins and CVD are null, leading to the conclusion that vitamins have no role in CVD prevention. Our objective was to examine challenges inherent in single nutrient trials using vitamin D and CVD as a case study.

**Methods:**

We conducted a systematic scoping review of the literature published since 2011 on vitamin D and CVD, including RCTs, prospective cohort studies (PCS) and systematic reviews from Medline, Web of Science and Cochrane. Studies were conducted in adults and included CVD outcomes with a minimum sample of 500 for RCTs and 10,000 for PCS. We applied Bradford Hill criteria for the establishment of causality in biological systems.

**Results:**

The search yielded 4170 papers, of which 40 were eligible, including 6 RCTs and 7 PCS. The Bradford Hill analysis of a causal relationship between vitamin D and CVD was mixed, with strong mechanistic support and reasonable strength and consistency in observational data but weak evidence of temporality. There was sufficient justification for trialling a benefit for CVD prevention but RCTs were inconsistent with the mechanistic and observational studies and reported mostly null results. Contextual factors were key, including baseline vitamin D status among participants, background supplementation and underlying participant disease profiles.

**Conclusion:**

This example illustrates the complexity of conducting nutrient trials and raises questions about RCTs of single nutrients for complex chronic diseases. The core challenge common to all nutrient trials is the absence of a zero-intake placebo group and variable background exposures. Alternative approaches and interpretation paradigms are required.

**Supplementary Information:**

The online version contains supplementary material available at 10.1007/s00394-025-03706-w.

## Introduction

Many well-designed, large-scale randomised controlled trials (RCT) of nutrients and chronic disease prevention have reported null or inconclusive results. In this systematic scoping review, we discuss some of the challenges inherent in single nutrient trials using the example of vitamin D and cardiovascular disease (CVD).

### Historical context

Since the 1940 s, evidence-based medicine has relied on the double-blind, randomised, placebo-controlled trial as the gold standard for generating evidence of causality, meaning that the intervention (be it a drug, device, vaccine or surgery) directly caused the outcome (cure, reversal of symptoms or prevention of disease). Dietary interventions are among the first RCTs on record [1]. Although infectious disease theory was dominant around the turn of the twentieth century, the identification of non-infectious causes of diseases such as xerophthalmia, pellagra and rickets led to a series of landmark discoveries of dietary components that were essential for disease prevention. A combination of results from direct observation, dietary interventions, mechanistic studies and chemical analysis revealed the structures, functions and deficiency symptoms of several vitamins and formed the basis for establishing early dietary reference values for prevention of vitamin deficiencies [2].

Early dietary recommendations for the prevention of vitamin D deficiency in children were strongly influenced by intervention studies on nutritional rickets in Vienna led by Chick and colleagues [3], who reported prevention and reversal of rickets among young children with the addition of 6–8 g cod-liver oil to their diet. In prevention studies, X-ray confirmed the absence of rickets before randomisation; intervention and control diets were standardised; UVB exposure levels were controlled; and outcomes were prespecified and clinically verified. Findings were supported by animal studies of experimental rickets [4] and were consistent with other data (reviewed by [5]). These studies benefited from what Heaney [6] referred to as a neat “index mechanism”; in the case of the Vienna studies, malabsorption of calcium and phosphorus due to vitamin D deficiency causing rickets was reversed by adding vitamin D to food, and rapidly demonstrated by the “short latency” of the disease. The Vienna studies also complied with many of the criteria proposed by Lappe and Heaney [7] for an RCT to be informative, including the use of a true control group with zero or very low exposure, an intervention dose sufficient to separate the treatment and controls, standardised outcomes and optimisation of the co-nutrients (e.g. calcium).

### Beyond clinical deficiency to prevention of non-communicable disease

Nutrients are multi-organ and multi-system agents due to their influences on all physiological processes, maintaining body function, enabling growth and development and moderating ageing-related decline. No nutrient has a single target cell, metabolic pathway or function and many of them interact with each other metabolically e.g. vitamin D and calcium, B-vitamins in one-carbon metabolism. Many effects of vitamin D are exerted through the actions of its active metabolite, calcitriol (1, 25(OH)_2_D), which interacts with the vitamin D receptor (VDR), a ligand-activated transcription factor present in most tissues, to control gene expression [8]. Considerable laboratory evidence exists of the cellular effects of calcitriol on immune regulation, apoptosis, autophagy, inflammation and differentiation [8, 9]. As calcitriol availability depends on its precursor, 25-hydroxyvitamin D (25(OH)D), the biomarker of vitamin D exposure from UVB exposure and diet, it might be anticipated that nutritional status of vitamin D would influence the ability of calcitriol to exert its effects, with consequent health outcomes. Mechanistic, preclinical and clinical studies link vitamin D to CVD, including modulation of endothelial function, hypertension, systemic and vascular inflammation [10]. The risk of CVD is higher among people with type 2 diabetes and vitamin D supplementation has been shown to decrease the risk for developing diabetes among people at high risk [11]. Other nutrients have also been shown to interact with vitamin D, e.g. Fe [12, 13], Mg [14] and Zn [15], which illustrates the complexity of exploring nutrition and disease outcomes and the interdependency of vitamin D with other nutrients.

There is a high prevalence of low vitamin D status in many regions; risk factors include geographical location, lack of UV exposure, older age, dark skin pigmentation, diet, adiposity, smoking, social deprivation and female sex [16]. Many large prospective cohorts associate low vitamin D status with increased risk of important health outcomes, including CVD, cancer and mortality [17–20]. Meta-analyses of prospective studies, such as Wang et al. [21], have reported inverse associations between circulating 25(OH)D and CVD risk. However, observational studies are subject to confounding and many of the risk factors for CVD are the same as those for low vitamin D status. Some Mendelian Randomisation (MR) studies have reported increased CVD among those with genetically low vitamin D status (25(OH)D < 50 nmol/L) [22], a threshold which includes ~ 40% of the EU population [32]. However, a reanalysis of the work by Zhou et al. [22] in the UK Biobank accounting for heterogeneity in the genetic effects on serum 25(OH)D has indicated that this association no longer stands [24]. Evidence-based medicine (and evidence-based nutrition) relies mainly on RCTs, and to date, outcomes from most RCTs have not reported a benefit of supplementation with vitamin D for CVD prevention [25–28].

### Why do observation studies frequently not translate to positive outcomes in RCTs?

Many RCTs of nutrients and CVD have delivered outcomes that are inconsistent with observational data, including vitamins E, C and B-vitamins [29–32] and some of the reasons why nutrient RCTs are distinct from medical trials are acknowledged [27]. These include the impossibility of a true, zero-dose placebo control, especially in the case of vitamin D with dual exposure modalities, and the difficulties of assessing daily variations in the background exposures of both treatment and control groups. Several investigators have produced thoughtful analyses of their RCT data for scientific, nutrition, and medical communities to consider, which may help minimise future errors and research redundancy [33, 34]. In the context of health promotion and disease prevention, is it feasible that a nutrient such as vitamin D, provided at a dose exceeding habitual intake over a defined time-frame, usually < 5 years, could prevent or reverse a disease that develops over a long time? If it is unfeasible that a nutrient could behave like a therapeutic agent, should we also question whether trials of nutrients, which have wide safety margins and are consumed habitually, be designed and evaluated differently than proprietary pharmaceutical agents?

The objective of this systematic scoping review was to evaluate the current RCT-centred approach to investigating the role of nutrients in chronic disease prevention. Using the example of vitamin D and CVD, we conducted a narrative evaluation of the available evidence from large-scale RCTs and applied Bradford Hill criteria to evidence for the vitamin D-CVD hypothesis, including prospective cohort studies as supporting data. We consider the challenges of using single-nutrient RCTs to investigate CVD causality and consider whether strategies can be developed to address common issues. This review is part of a broader collaborative effort by the Federation of the European Nutrition Societies to improve standards in the science of nutrition [35], under working group one, focusing on concepts and methodologies.

## Methods

A systematic scoping review was conducted, given the aim of exploring the conceptual boundaries of the topic [36].

The following systematic electronic search strategy was developed and performed.

### Search criteria

Search terms were as follows:

Vitamin D/exp OR Cholecalciferol OR calcidiol OR 25-hydroxyvitamin D AND vitamin D (and other terms) supplementation AND cardiovascular disease OR coronary heart disease OR atherosclerosis OR stroke OR cerebrovascular accident AND randomized controlled trial OR clinical trial OR cohort OR dietary intervention OR meta-analysis OR systematic review. These (or adapted search terms depending on the database) searches were conducted within the Medline (National Library of Medicine, Bethesda, Maryland, MD, USA), Web of Science and Cochrane Register of Controlled Trials (The Cochrane Collaboration, Oxford, UK) databases. The searches were conducted in January 2023. References were initially imported into Endnote reference management software from the different databases and search results combined. Duplicates were removed. Papers were then submitted to Rayyan for double-blind evaluation of suitability by two reviewers (JW and either TD or GT) according to the eligibility criteria. Abstracts were initially screened, followed by full text screening of remaining papers. Any conflicts were identified following unblinding, discussed and consensus achieved. The references of the identified studies and review articles were scrutinised to look for any additional studies of interest. An informal literature search conducted in June 2024 ensured that all recent relevant references since the previous formal search or by article alerts which followed had been captured.

The following eligibility criteria were applied:

- Studies were in human adults (> 18 years) and published in English within 10 years of the first search (from 2011 onwards).
- Studies were RCTs of vitamin D alone with CVD-relevant endpoints, or systematic reviews/meta-analyses of these trials, or prospective cohort studies or systematic reviews/meta-analyses of these cohorts.
- Studies had a minimum sample size of n = 500 participants for RCTs or n = 10,000 participants for prospective cohort studies.
- Prospective cohort studies considered separately (i.e. not within meta-analyses and/or systematic reviews) reported clinically validated outcome assessments (e.g. CVD, coronary heart disease, myocardial infarction, stroke, CVD-related mortality) and quality assured analytical methods for circulating 25(OH)D.

Bradford Hill criteria were used to evaluate the likelihood of a causal association between low vitamin D status and increased risk of CVD outcomes by applying each of the Bradford Hill criteria for causality in a biological system [37], based on the eligible trials, systematic reviews/meta-analyses and cohorts. The applicability of these criteria to nutrient trials was considered alongside other analyses of the evidence for nutrients, bioactives and health outcomes [38, 39]. Bradford-Hill criteria had previously been applied to this question prior to the conduct of most large RCTs [40] and are still considered to be relevant [41].

## Results

### Literature review

**Fig. 1:**
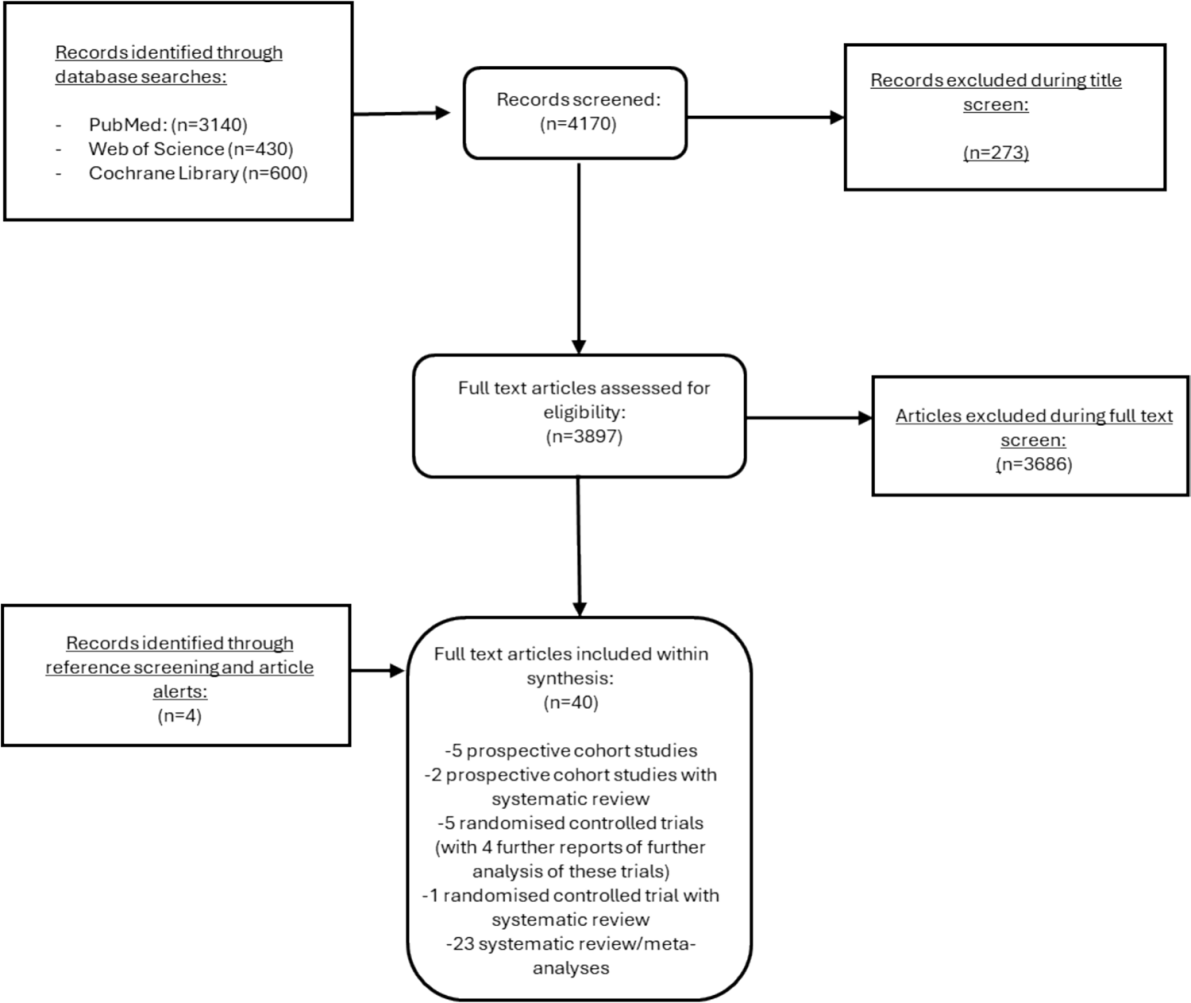
PRISMA flow chart summarising study selection; the eligible papers are listed within Supplementary Table 1

The initial searches after removal of duplicates identified a total of 4170 potentially eligible papers. The PRISMA flow chart is illustrated in Fig. 1. After screening at abstract and full text stage, with consideration for eligibility, a final total of 36 studies were included; an additional four studies were identified from reference lists or from article alerts from databases after the formal search, leading to a total of 40 studies. Relevant papers included RCTs, which were the main focus, including publications of primary and secondary outcomes, prospective cohort studies and systematic reviews, which were used to assess some of the Bradford Hill criteria. Some systematic reviews considered only RCTs or observational studies and some considered both; the inclusion criteria for cohort studies, as detailed in methods, would have been different to what was applied when eligible study selection was occurring within published systematic reviews.

### Bradford-hill analysis of observational studies

The outcomes of the analysis according to Bradford-Hill criteria for observational studies are summarised in Table 1. Where required to interpret the findings for these criteria in detail, additional studies were included. For one study arising from the literature search the paper has been retracted and republished [42, 43]; therefore, the corrected version has been included [43].

**Table 1:** Summary of Bradford Hill criteria for a causal relationship between Vitamin D status and CVD outcomes and established markers of risk

	Strength of association	Consistency	Temporality	Specificity	Biological gradient	Plausibility	Coherence	Experiment	Analogy
Outcomes of interest									
CVD mortality	Yes^a,c^	Mostly^c^	Maybe^c,d^	No^e^	Yes^f^	Yes^g^	Yes^h^	No but has the hypothesis been tested	Yes
Stroke	Yes^b,c^	Mostly^c^	Maybe^c,d^	No^e^	Yes^f^	Yes^g^	Yes^h^	No but has the hypothesis been tested	Yes
Myocardial infarction/ CHD	Yes^b,c^	Mostly^c^	Maybe^c,d^	No^e^	Yes^f^	Yes^g^	Yes^h^	Possibly	Yes
All-cause mortality	Yes^c^	Yes^c^	Yes^c,d^	No^e^	Yes^f^	Yes^g^	Yes^h^	Yes, among older adults	Yes

^a^At higher concentrations association null (ERFC)

^b^No strong association when baseline 25(OH)D above 50 nmol/l

^c^Observations are not 100% consistent but predominantly consistent associations reported. Inconsistency observed may be due to baseline vitamin D status of population and variability in follow-up time. Impact of ethnicity and sex may be important, although this has not been widely examined

For the cohort studies, the data provides sufficient evidence that the criterion of temporality is met. For RCTs some participants had CVD events before enrolment

^d^Temporality observed, however, incipient CVD at baseline not assessed, therefore criterion difficult to be met by prospective cohort studies

^e^Nutrients, including vitamin D, rarely have single specific effects therefore this criterion may be less relevant for nutrition science

^f^Recent analyses suggest associations may be non-linear; not explored in earlier analyses

^g^Biologically plausible mechanism demonstrated for hypertension, thrombogenesis, endothelial function, infection/inflammation

^h^Lack of coherence across different study designs; but coherence with known biological facts present

### Strength of association and biological gradient

The strength of the association between 25(OH)D concentrations and a range of cardiovascular (CV) outcomes, including overall CVD, as well as MI/CHD and stroke was assessed by Sofianopoulou et al. [43] who undertook a dose–response analysis of the relationship between 25(OH)D concentrations and risk of coronary heart disease, stroke, and all-cause mortality, using data from 33 prospective cohort studies. Collectively, these studies comprised 500,962 participants who had no known history of CHD or stroke at baseline. At low concentrations of 25(OH)D, there were inverse non-linear associations with incident CHD, stroke and all-cause mortality. Other meta-analyses have focused on older adults, finding an increased risk of CVD mortality in those with 25(OH)D concentrations < 50 nmol/l (RR1.54; 95% CI 1.24–1.91) [44]. Using individual participant data from a European consortium with standardised measurement of serum 25(OH)D and CV outcome specification, Gaksch et al. [45] reported a higher risk (HR 2.21; 95% CI 1.50–3.26) of death from CV causes for those with 25(OH)D concentrations < 30 nmol/L, compared to the reference group of 75–99.99 nmol/l). These data suggest strong associations between low vitamin D status and CVD outcomes including CV mortality.

### Consistency of association and temporality

Evaluating CVD outcomes by differing study designs, analytical methods and cut-points for 25(OH)D, population characteristics and statistical approaches can help to determine consistency. As temporality refers to the concept that the exposure must precede the onset of the outcome of interest, prospective cohort studies offer evidence that the criterion of temporality is met. Reported associations with 25(OH)D appear to be consistent across CVD sub-types [43, 46]. Baseline vitamin D status affected the association with outcome [45], therefore inconsistencies may relate to the vitamin D status of the population being considered. For example, Yang et al. [44] found significant heterogeneity in their meta-analysis of vitamin D and CVD outcomes in older adults (P = 0.004), yet this heterogeneity was removed when they only included those in the analysis who had circulating 25(OH)D < 50 nmol/L. Yang et al. [44] also found that study quality and overall study population size did not seem to influence the heterogeneity of the findings, although studies with case numbers >  = 200 observed more homogeneous outcomes. Zhang et al. [47] found that the longer the follow-up time, the lower the benefit of higher baseline 25(OH)D concentrations.

The use of race and ethnicity as proxy indicators of skin tone affect the interpretation of associations between vitamin D status and CVD [48], as disease risk profiles vary by context and socio-economic status and light skin tone is generally associated with higher circulating 25(OH)D. This is demonstrated by Robinson-Cohen et al. [49], who reported an inverse association with vitamin D status and incident CHD events among participants who were white or Chinese but not black or Hispanic, although this study did not meet our formal criteria for inclusion as numbers were below the n = 10,000 inclusion criteria cut-off. Clear definitions of race distinct from skin pigmentation, as well as consideration of cultural skin-covering clothing use, are required to detangle interactions of vitamin D and disease outcomes among people of different race, ethnicity and socio-demographic backgrounds [8].

Overall, observational studies often report an association between circulating 25(OH)D and CV outcomes, with inconsistencies possibly related to population vitamin D status, duration of follow up specification of outcome or lack of specificity for ethnicity or skin tone.

The temporality criterion is observed between lower 25(OH)D and higher risk of CVD in prospective cohort studies, however, preclinical CVD and risk factors for incipient diseases are not assessed at baseline, therefore this criterion cannot be regarded as fully met (Table 1).

### Specificity

The specificity criterion explores to what extent the exposure of interest is linked to a specific outcome. Many nutritional factors, including vitamin D, have diverse effects and influence multiple outcomes. In their comprehensive review of the evidence for causal effects of dietary factors on cardiometabolic diseases, Micha et al. [38] applied Bradford-Hill criteria to estimate dose–response effects of consumption of 17 individual foods and nutrients for which there was compelling evidence. Specificity was evaluated on three principles: 1) dietary factor influences a mechanism or pathways known to cause the outcome; 2) dietary factor not associated with multiple other, unrelated non-communicable diseases (e.g. multiple cancers); 3) dietary association has additional specificity within the set of cardiometabolic outcomes (coronary heart disease (CHD), stroke, diabetes mellitus). While vitamin D does influence mechanisms and pathways known to cause CV outcomes, low serum 25(OH)D has been linked to risk of a range of other diseases and the association of vitamin D with CVD does not have additional specificity within the outcomes considered. Therefore, this Bradford-Hill criterion may have little relevance for studies of individual nutrients, as none have a single effect or have been linked to a single outcome. Indeed, a previous evaluation of Bradford-Hill criteria to the vitamin D and CVD topic [40] did not include this criterion for assessment.

### Plausibility

Plausibility of a role for vitamin D in CVD prevention centres on its effect on endothelial and smooth muscle cell function; direct effects on cardiomyocytes; immune function and the cardiovascular system, including underlying pathologies such as hypertension, inflammation, impaired glucose metabolism and type 2 diabetes as well as possibly lipid metabolism (reviewed by [8, 50]). Effects on endothelial cells are mediated by nitric oxide (NO) synthesised from L-arginine and NO synthase (eNOS), which is Ca-dependent. 1,25(OH)_2_D is required for cellular Ca uptake in the epithelium and VDR knockout mice are characterized by a reduction in eNOS expression, as well as an impaired response to acetylcholine induced vasorelaxation in the aorta [51]. In vitro, endothelial cells treated with 1, 25(OH)_2_D show increased NO production and abundance of NO synthase, and these effects are not detected in absence of the VDR [52]. Direct effects of vitamin D depletion or VDR deletion have been demonstrated in vitro and in pre-clinical studies; for example, deletion of the VDR in cardiomyocytes causes myocardial hypertrophy and fibrosis [53] and vitamin D deficiency in chicks and rats produced myocardial dysfunction [8].

Effects of vitamin D on the cardiovascular system, have focussed mainly on hypertension, inflammation and, more recently, lipids. Briefly, the kidney cells responsible for renin production (juxtaglomerular cells) are sensitive to calcitriol, and VDR and CYP27B1 knock-out mice develop high renin systemic hypertension [54] which can be rescued with angiotensin converting inhibitors. A promotor region for the VDR element has been identified in the promoter region of the renin gene, suggesting that renin is directly regulated by calcitriol. Although not all studies identified an increase in renin concentration in VDR null mice, high thrombogenesis and lower fibrinolysis was identified in these animals [55]. The VDR is present in all immune cells and 1, 25(OH)_2_D regulates the inflammatory profile, decreasing pro-inflammatory (e.g. IL-1, IL-6) and up-regulating anti-inflammatory cytokines (e.g. IL-10), as well as chemokines and other signalling molecules, which influence vascular function. Effects on lipid metabolism are complex and confounded in clinical studies by adiposity and medications and Mendelian randomization studies have yielded conflicting results [56].

### Coherence

While coherence with known biological facts and plausible biological mechanisms is present, together with compelling observational data, there is a lack of coherent evidence across different clinical study designs, i.e. the lack of coherence across longitudinal cohort studies and RCTs is the focus of the current discussion. We consider that coherence between the biological and epidemiological evidence is present.

### Analogy

Analogy describes the consistency of findings in terms of related associations. Sunlight exposure causes an increase in Vitamin D status, and thus is analogous to increased dietary or supplemental Vitamin D intake, yet sunlight exerts effects on relevant outcomes may be unrelated to vitamin D. Pre-Western Inuit people have historically had a low occurrence of CHD and this has been linked to a traditional diet high in wild fish and marine mammals, which are rich sources of protein, vitamin D and omega-3 fats and low in refined carbohydrates. In common with many communities in transition, the occurrence of CHD among Inuit in Greenland has increased, tracking changes in diet and lifestyle, including a reduction in the dominance of traditional diets and increases in purchased food items, with lower intakes of vitamin D, marine oils and higher intakes of high fat, salt and sugar-containing foods [57]. The previous evaluation of the link between vitamin D and CVD using Bradford-Hill criteria [40] considered analogy in terms of the association between vitamin D and other conditions, e.g. cancer, but we do not consider that to be the most appropriate application of the criterion.

In summary, the Bradford-Hill analysis of a causal relationship between vitamin D and CVD is mixed; with strong mechanistic support in the plausibility domain, reasonable strength and consistency in observational data, but weak evidence of temporality and no consistency of effect, as the biological effects are exerted across cardiovascular tissues and associated systems.

### Experimental: randomised controlled trials

The characteristics of the five RCTs included in this analysis [58–62] are described in Table 2. Studies were conducted in high-income settings and included > 60,000 participants in total, with sample sizes from 2495 to 25,871. Participants were all at least 50 years of age and there was a fairly even gender balance in four out of five studies. All studies used vitamin D_3_ and doses ranged from daily doses of 800IU (20 μg) in RECORD [62] and 2000 IU (50 μg) in VITAL [60], to monthly doses of 60,000 IU (1500 μg) in D-Health [58] and 100,000 IU (2500 μg) in VIDA [61], following a loading dose. FIND was the only dose–response intervention, albeit with only two doses, i.e. daily doses of 1600 IU (40 μg) or 3200 IU (80 μg) [59]. All studies used a blank placebo control and permitted participants to continue taking their own vitamin D supplements in doses from 200 to 800 IU per day. Adherence to supplementation as well as potential contamination were monitored in all trials.

**Table 2:** Summary of Randomised Controlled Trials of Vitamin D and Cardiovascular Disease with sample sizes > 500

Author	Neale et al	Virtanen et al	Manson et al	Scragg et al	Avenell et al
Year	2022	2022	2019	2017	2012
Acronym	D-Health	FIND	VITAL	VIDA	RECORD
Country	Australia	Finland	US	New Zealand	UK
Sample	21,315	2495	25,871	5110	5292
Population	General	General	General	General	History Fracture
Age range	60-84y	>/= 60y M; >/= 65y F	>/= 50y M; >/= 55y F	50-84y	> 70y
% Female	46%	43%	51%	42%	85%
Treatment	D3 60000IU	D3 3200IU or 1600IU	D3 2000IU	D3 200000IU bolus followed by 100000IU	D3 800IU
Frequency	Monthly	Daily	Daily	Monthly	Daily
Control	Blank placebo	Blank placebo	2X2 design; DHA + EPA 840 mg, blank placebo	Blank placebo	2 × 2 design; Ca 1000 mg, blank placebo
Duration	5.4–7.7y	5y	5.3y	2.5–4.2y	2–5.2y
Primary CV outcomes	CV Mortality	Major CVD event (composite MI, stroke or CVD mortality)	Major CV events	CVD mortality	CV mortality
ITT Relative Effect (95% CI)	HR 0·96 (0·72–1·28)	3200IU HR 0.84 (0.54–1.31); 1600IU HR 0.97 (0.63–1.49)	HR 0.97 (0.85–1.12)	HR 1.02 (0.87–1.20)	HR 0.91 (0.79–1.05)
Secondary CV outcomes	MI, Coronary revascularisation, Stroke	MI, stroke, CVD mortality	MI, stroke or CV mortality composite outcome	MI, HT, Angina, Heart failure, Stroke, All CVD	Cardiac failure, MI, stroke, composite outcome
Secondary CV effect	MI: HR 0.81 (0.67–0.98)	No effect	No effect	No effect	Cardiac failure: HR 0.75 (0.58, 0.97)
CV conditions at baseline	Hx CVD 22%; CVD drug 46%; statin 35%	42% BP-lowering; 29% statin; 9% diabetes	50% BP-lowering + 38% chol-lowering; 45% aspirin; 14% diabetes	6% MI, 37% HT, 10% Diabetes	8% diabetes
Baseline 25(OH)D *as reported*	Predicted 25(OH)D estimated 24% < 50 nmol/L	Measured subgroup mean (SD) 75 (18) nmol/L	Measured subgroup mean (SD) 30.8 (10) ng/mL	Measured mean (SD) 24.4 (9.6) ng/mL	Measured subgroup < 38 nmol/L
Ongoing vitamin D supplement	Up to 500IU/day	Up to 800IU/day	Up to 800IU/day	Up to 600IU 50-70y Up to 800IU 71-84y	Up to 200IU/day
Follow-up	None to date	None to date	None to date	None to date	3 years
Selected publications	Thompson et al. 2023	Virtanen et al. 2023	Manson et al. 2020	Scragg et al., 2019; 2020	Ford et al. 2014

*y* Year, *M* Male, *F* Female, *CV* Cardiovascular, *MI* Myocardial infarction,* ITT* Intention to treat, *CI* Confidence interval, *HR* Hazard ratio, *HT* Hypertension, *25(OH)D* 25-hydroxyvitamin D, *BP* Blood pressure, *Chol* Cholesterol; multiply 25(OH)D in ng/mL by 2.496 to convert to nmol/L

After follow-up periods of between ~ 2.5 and ~ 7.5 years, primary cardiovascular outcomes were CV mortality (RECORD, D-Health, VIDA) [58, 61, 62] and major CV events (VITAL, FIND) [59, 60]. Secondary outcomes included myocardial infarction (MI) and stroke (all studies) [58–62]; cardiac failure (RECORD, VIDA) [61, 62] and composite CV outcomes. Outcomes were verified by linking to a combination of hospital admissions, health insurance and mortality data (D-Health) [58], physician-verified self-reports (VITAL) [60], electronic health records (VIDA, FIND) [59, 61] or mortality data (RECORD) [62]. Participants had various background prevalence rates of CV-related conditions, including type-2 diabetes. Hypertension and medication use were recorded by most studies to verify the presence of underlying conditions. Where baseline medications were recorded, 30 to 50% of participants were taking cholesterol and blood pressure-reducing medications, respectively.

All five RCTs reported primary outcomes with hazard ratios at or close to 1. There were benefits of vitamin D supplementation for some secondary outcomes and secondary analyses continue to be published, as well as analyses of pre-specified outcomes across the trials [63]. In a detailed analysis of pre-specified outcomes in D-Health, Thompson et al. [64] reported that the rate of MI was lower (HR [CI] 0.81 [0.67–0.98]) in the vitamin D group (Table 2) than in placebo. This was similar in the VIDA trial, albeit with a wider confidence interval (0.90 [0.54–1.50]) [61]. Considering baseline characteristics of participants, Thompson et al. [64] reported that the HR of major CV events was lower in people using statins and other CV drugs at baseline versus those who were not. This might be an important finding, particularly considering the proportion of the older adult population living with heart disease [65].

Baseline analysis of circulating 25(OH)D) was completed in VIDA [61] and among subgroups of RECORD [62], VITAL [60] and FIND [59] and was predicted in D-Health [58]. Except for RECORD [62], where the measured subgroup had a 25(OH)D concentration < 38 nmol/L, participants had mean or estimated mean 25(OH)D around 25–30 ng/mL (~ 70 nmol/L). This is similar to recent estimates from the United States among adults over 50 years [66] and higher than data from national surveys and adult cohorts in Europe [23]. Low vitamin D status (25(OH)D < 50 nmol/L) was reported among 13% in VITAL [60]; 9% in FIND [59] and 30% in VIDA [61], with very few participants below 25 nmol/L [63]. Studies were not designed or powered to support subgroup analysis and neither VITAL [60] nor VIDA [61] reported any differences in hazard ratios for CV events among participants with baseline 25(OH)D < 50 nmol/L.

All of the RCTs discussed were implemented in high-income settings. A further trial, the International Polycap Study (TIPS), used a 2 × 2 × 2 factorial design to investigate the effect of an experimental capsule containing four cardioprotective medications or low-dose aspirin or vitamin D (60,000 IU/month) versus placebo on CVD outcomes [67]. This study was conducted among 5670 adults, predominantly drawn from South Asia, South East Asia and South American countries. Participants were > 50 years with elevated CVD risk. The vitamin D factorial was powered for fracture, but reported a composite CV outcome, which was not significantly different between intervention and control. This trial was severely disrupted due to the COVID-19 pandemic.

## Discussion

This systematic scoping review of observational and experimental data aimed to evaluate the RCT-centred approach to investigating the role of nutrients in chronic disease prevention, using the example of vitamin D and CVD. The key question is whether a vitamin D supplement, provided at a dose exceeding habitual intakes over 2 to ~ 7 years, could prevent CVD, including cardiovascular mortality and major events. We applied Bradford-Hill criteria to evidence from prospective and laboratory studies and concluded that the criteria of biological plausibility, strength and, to a lesser extent, consistency of the association with CV outcomes were met, providing adequate justification for considering vitamin D as a causative agent. The mechanistic and epidemiological data are reasonably coherent and evidence for temporality is present from prospective observational cohort studies. We focused on five recent high-quality large-scale trials to evaluate the experimental criterion and for our purposes, the RCT-centred approach to nutrient research. The outcomes from these trials are inconsistent with the mechanistic and observational data and have largely failed to reject the null hypothesis, except for some secondary, albeit important outcomes.

A decade ago, Weyland et al. [40] completed a similar analysis with some differences; the authors did not use a systematic search strategy and four out of five of the vitamin D RCTs available to us were not yet published. At that time, the experimental criterion was largely evaluated with trials that measured risk factor endpoints and the conclusion was that all relevant criteria for a causal association in a biological system were satisfied to indicate low 25(OH)D as a CVD risk factor.

Despite the large and excellent body of research now available, with five high-quality studies among ~ 60,000 individuals, we are unable to conclude whether vitamin D has a role in CVD prevention. In the VITAL, VIDA, D-Health and FIND studies [58–61], the majority of participants had relatively high baseline circulating 25(OH)D, relative to public health guidelines. This was highlighted in the Endocrine Society’s recent Clinical Practice Guideline on vitamin D for the prevention of disease [68], which was supported by systematic reviews of RCTs for each question addressed [28]. Based on the evidence available, which included the RCTs under consideration here, the SRs concluded with high certainty that vitamin D supplementation did not reduce the risk of cardiovascular events, stroke or MI among adults between 50 and 74 years. The guideline panel highlighted the difficulty of evaluating the efficacy of RCTs among participants with baseline vitamin D status that may be considered healthy relative to most international public health recommendations, noting that the lack of effect of vitamin D did not necessarily indicate that vitamin D does not influence the relevant outcome, but rather that the study populations had adequate status for the desired outcome, before supplementation [68]. Therefore, the hypothesis that vitamin D supplementation *might* have CV benefits among individuals following correction of low vitamin D status is largely reliant on the RECORD trial [62], which was not a priori designed to evaluate secondary CV outcomes. On this basis, it seems most fair to conclude that the hypothesis has not been fully tested. This systematic scoping review could have included selection of participants with an appropriate baseline vitamin D status as an additional inclusion criterion, but that would have removed many of the included studies.

In RCTs of nutrients, dietary intakes should ideally be standardised across treatment groups [7]. While this would not be feasible in a super-trial lasting many years, it has to be acknowledged that the RCTs analysed did not account for background vitamin D intakes or food sources of vitamin D or calcium intake. Even more importantly, they did not include a true control (i.e. zero or low exposure), as, while these were placebo-controlled trials, significant numbers of participants in both treatment and placebo groups continued to consume their usual vitamin D supplements, including doses up to 800IU, which together with background dietary intake would deliver at least 1000 IU (25 μg) of vitamin D, a dose that exceeds current recommendations for vitamin D intake in major global regions, and is known to prevent low vitamin D status (25(OH)D < 50 nmol/L), regardless of UVB exposure.

The treatment doses for vitamin D supplementation were highly variable across the trials. It may be worth considering the potential of a more personalised approach where the dose is tailored to an individual’s baseline status and adapted to achieve hypothetical status thresholds, such as that used by Aloia et al. [69]. Repeated measures of 25(OH)D, including the vitamin D metabolome, would permit dose adaptation. Development of such approaches would also address concerns around differential metabolism and dose–response of vitamin D among individuals based on individual characteristics, such as sex, age, adiposity and genotype [70]. Continuous improvements in blood spot analytical technology may enable remote monitoring and an agile adaptation of supplementation.

Lappe and Heaney [7] also recommended that the study population should be disease-free at the outset of a trial. As would be expected among older adults, the RCTs of vitamin D and CVD included a significant proportion of individuals with underlying CVD, who were being managed for hypertension, type 2 diabetes and dyslipidemia, meaning that participants had heterogenous disease risk profiles. The RCTs considered were designed and initiated over the past 10–15 years. Clinical management of CVD is evolving quickly, with risk factor identification and pharmacological prevention methods well-advanced in the regions where the studies were implemented. Declining CV mortality is well-documented [71], largely attributed to a combination of primary prevention, including lifestyle and clinical risk management, early diagnosis and acute care [72].

In this context, current clinical management of those at high risk of CV outcomes needs to be considered as RCT findings are interpreted. The most recent analysis of D-Health [64] suggested that the rate of major events was lower in the treatment group, particularly among those who were taking risk-modification drugs at baseline. To our knowledge, this is the first time such an analysis has been published, and the authors call for further evaluation of the role of vitamin D supplementation among people on drug treatment for prevention or treatment of CVD. Robien et al. [73] suggested that interactions between vitamin D and medications metabolised by cytochrome P450 3 A4 (CYP3 A4) are particularly important as vitamin D-statin interactions vary by drug. Such interactions also have implications for the duration of the intervention. The duration of the RCTs considered here varied considerably, particularly relative to the likely period of disease development, and while intervention studies can improve 25(OH)D status, it is unlikely they could impact CVD risk in the short term. Community agreement is needed on the optimal duration of supplementation for specific disease risk factors. Studies powered sufficiently to enable stratified analysis by risk factor, or a combination of risk factors and treatment modes could be of huge benefit as the reliance on cardiovascular mortality as an outcome becomes less relevant. It is worth highlighting the benefits observed in reducing progression to type 2 diabetes from vitamin D supplementation among individuals at high risk for diabetes [11, 28], which is likely to have downstream benefits for CVD progression among beneficiaries.

In the century since most of the fat-soluble vitamins were described, significant advances in the field of nutrition science have been made. The agility of the field is clear; a pertinent example is the transition from a reductionist approach focusing on one nutrient to the recognition of the importance of dietary patterns [74]. Notwithstanding our professional bias, adequate consideration of nutrition science in RCTs of nutrients and disease outcomes would help to offset some of the risks inherent in the core assumption of most nutrient studies, including vitamin D and others, that nutritional supplements are equivalent to drugs. Clearly, this is not the case. Consistently with vitamin D, the antioxidant vitamins (C, E and beta-carotene) and the B vitamins (folate, B6 and B12) showed initially promising associations in large cohorts, which were followed by large-scale RCTs with negative or inconclusive outcomes. The observational analyses have been reviewed [29, 30], and RCT data have been summarised in systematic reviews and meta-analyses [31, 32]. The data have also been synthesised in reviews considering supplemental approaches to reduce the risk of CVD [75–77]. Most recently, it has been concluded that the antioxidant vitamins show no consistent benefit for the prevention of CV disease, MI, or stroke following single nutrient trials, nor is there a benefit for all-cause mortality [78]. Preventive benefits for stroke from both folic acid and other B vitamins have been reported, with evidence graded as moderate quality, but not for other outcomes [78]. While accurate measurement of habitual diet is challenging, authors conclude that dietary risk factors in high-income countries are prevalent, but conclusive evidence for the benefit of supplements across different dietary backgrounds has not been demonstrated. Unsurprisingly, particularly against a background of prevalent obesity, vitamin supplementation is not a substitute for a healthy diet and is unlikely to overcome the adverse metabolic and nutritional consequences of excess adiposity.

Such a conclusion closely aligns with that suggested by Forman and Altman in 2004 [79] in an editorial for one of the first Cochrane reviews considering the potential role of antioxidants in cancer prevention, “the effect on diseases with long latency periods of pharmacological doses of specific micronutrients over a few years in middle-aged adults is a different scenario from physiological doses of the same micronutrients provided as part of a balanced diet on a lifelong basis, starting in childhood.” Our example demonstrates that, although there was sufficient justification for trialling a potential benefit for CVD prevention, the vitamin D RCTs conducted to date, despite excellence and care in design and implementation, have largely failed to test the underlying fundamental hypothesis. This does not affect the recognition that correction of vitamin D deficiency could benefit a condition arising from or exacerbated by that deficiency, but highlights the complexity of nutrients, where even the most rigorous studies need to be interpreted in the context in which they are conducted. Our analysis reveals the difficulty, and perhaps the impossibility, of trialling a single nutrient for chronic disease prevention, particularly in populations with a low prevalence of deficiency. Alternative methods and interpretation paradigms are required.

## Supplementary Information

Below is the link to the electronic supplementary material.

Supplementary file1 (XLSX 21 KB)

## Data Availability

No original data are presented in this study.
